# Development and Implementation of an End-Effector Upper Limb Rehabilitation Robot for Hemiplegic Patients with Line and Circle Tracking Training

**DOI:** 10.1155/2017/4931217

**Published:** 2017-06-15

**Authors:** Yali Liu, Chong Li, Linhong Ji, Sheng Bi, Xuemin Zhang, Jianfei Huo, Run Ji

**Affiliations:** ^1^Division of Intelligent and Biomechanical System, State Key Laboratory of Tribology, Tsinghua University, Haidian, Beijing, China; ^2^Rehabilitation Medical Center, Affiliated Hospital of National Research Center for Rehabilitation Technical Aids, Haidian, Beijing, China; ^3^Human Biomechanics Laboratory, National Research Center for Rehabilitation Technical Aids, Beijing, China

## Abstract

Numerous robots have been widely used to deliver rehabilitative training for hemiplegic patients to improve their functional ability. Because of the complexity and diversity of upper limb motion, customization of training patterns is one key factor during upper limb rehabilitation training. Most of the current rehabilitation robots cannot intelligently provide adaptive training parameters, and they have not been widely used in clinical rehabilitation. This article proposes a new end-effector upper limb rehabilitation robot, which is a two-link robotic arm with two active degrees of freedom. This work investigated the kinematics and dynamics of the robot system, the control system, and the realization of different rehabilitation therapies. We also explored the influence of constraint in rehabilitation therapies on interaction force and muscle activation. The deviation of the trajectory of the end effector and the required trajectory was less than 1 mm during the tasks, which demonstrated the movement accuracy of the robot. Besides, results also demonstrated the constraint exerted by the robot provided benefits for hemiplegic patients by changing muscle activation in the way similar to the movement pattern of the healthy subjects, which indicated that the robot can improve the patient's functional ability by training the normal movement pattern.

## 1. Introduction

Stroke is a leading cause of physical impairments, with symptoms of spasticity, weakness, and hemiplegia [1, 2]. Functional disability of upper limb is a common impairment among hemiplegic patients, which causes difficulties and inconvenience in activities of daily life [3, 4]. It has been reported that the repetitive interventions, such as constraint-induced movement therapy and variable task-oriented repetitive therapy, can improve movement coordination in patients with hemiplegic disabilities [5, 6]. Robots with its good repeatability and movement accuracy have been widely used in hemiplegic patients' physical therapy researches [6–9].

Although many robot rehabilitation therapies have been designed, such as the passive-guided mode, the active as needed, and the resistant mode [10–14], the clinical experiment with rehabilitation robots has not demonstrated expected effects, which may be caused by the patients' individual difference [15]. Researchers are paying more and more attention to improve the adaptation of rehabilitation robots to individual difference. Many researches have reported that researchers determine the equivalent impedance parameters of human upper limb online and offline by intelligent control algorithm to increase the adaptation of the robot system and improve the participants' experience [16–19]. Demir et al. [19] analyzed the patients' mechanical impedance parameters by neural network algorithm while training with their therapist and then used the parameters to activate the robot to imitate the interaction. Song et al. [20] developed an adaptive motion control for a 4-DOF end-effector upper limb robot based on impedance identification and confirmed that the control strategy can realize the adaption of the system among five healthy subjects' experiment. The intelligent control strategies have been considered as an effective method to improve the adaption of rehabilitation robot system during clinical experiment.

The parameters of movement therapy in most rehabilitation robots cannot be intelligently changed to individual difference. Because of the complex and diversity of upper limb movement during daily life, the clinical therapy for upper limb rehabilitation training should be customized. The rehabilitation robots based on neural networks have been widely discussed to change therapy parameters according to patients' conditions. Owing to the difficulty of intelligent control training and lack of sufficient training set [16, 20, 21], rehabilitation robots based on intelligent control strategies have been still not widely used in clinical rehabilitation.

The objective of this article is to develop a new rehabilitation robot based on interaction force and displacement of end-effector to help patients to train in point-to-point line and circle tracking tasks. Besides, this article provides some experiments to verify the effectiveness of the robot.

## 2. Materials and Methods

### 2.1. Description of the EEULRebot System

Upper extremity compound movement robot rehabilitation platform (UECM) was an end-effector rehabilitation robot providing trainings in a fixed plane [22, 23], which can only deliver passive-guided therapy for the patients with line and circle tracking tasks in clinical rehabilitation. End-effector upper limb rehabilitation robot (EEULRebot), the improved version of (UECM), is developed with multiple training modes for shoulder and elbow coordination for hemiplegic patients following a stroke. This new robot can train patients in multiple planes in order to imitate the activities of daily life, which is the improvement from UECM. When the deviation between actual movement and designed trajectory is larger than the threshold, or when the movement velocity is smaller than the threshold velocity which reflected patients' functional ability [24], training modes should be adjusted. The details of the adjustment are illustrated in the following section of control strategies, which will widen the robot application in clinical research.

Mechanical system of the EEULRebot is designed to mimic the human body structure, with two links similar to human upper arm and forearm. Besides, a handle and elbow support is implemented in the system, which is used to help patients hold their arms in normal posture.

**Figure 1:**
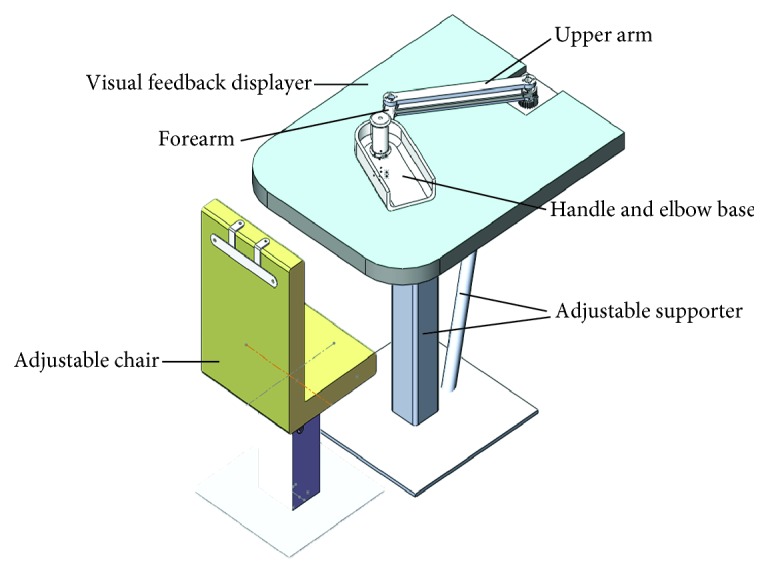
The Solidworks model of EEULRebot system.

EEULRebot is equipped with an adjustable height and inclination angle supporter actuated by a lift (LP2, LINAK, Denmark) as well as a height adjustable chair implemented by another lift (LB1, LINAK, Denmark). According to the patients' needs, the height of the supporter platform is in range of 700~1200 mm with the height of the designed chair in range of 350~750 mm. Besides, the planar inclination angle of the supporter platform is in range of −30°~60° for different planar training. Two Maxon RE40 DC motors are used to drive the upper limb and forearm, respectively, to realize the planar movement of end-effector. Two planetary gear reducer with a ratio of 53 : 1 (Maxon GP42) are used in order to increase the output torque of motors as well as decrease the output rotation speed. During one training, the end-effector is moved on one plane, and the planar force, which is a two-dimensional force, is needed to calculate the torque of each motor. So the robot is also equipped with a two-dimensional force sensor (BaiSen Instrument, China) that measures the interaction force between the human and the robot. The isometric view of the EEULRebot system is shown in Figure 1.

Besides the adjustable supporter system and the driving system, the EEULRebot system also includes a visual feedback displayer as a biofeedback system. This displayer will show the designed trajectory and the actual movement of the end-effector in different colors to highlight the difference, which will remind patients to adjust their movement to decrease the deviation.

### 2.2. Kinematic Model

**Figure 2:**
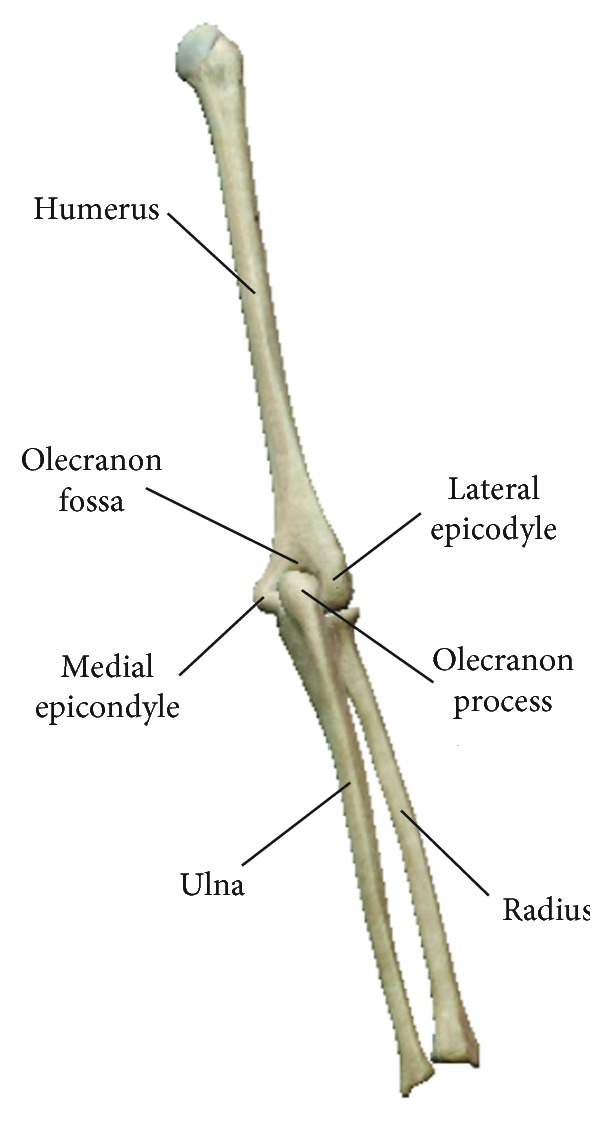
Posterior view of human upper limb. The humerus and ulna as well as radius form the elbow joint with their characteristic shape features: medial epicondyle, lateral epicondyle, olecranon fossa, and olecranon process.

The EEULRebot system has two serial links similar to human upper arm and forearm, which will have two different postures for a certain end-effector position. But, while we apply some anatomical features in human upper limb, we will get a determined solution for the two serial links inverse kinematics. Our actual forearm consisting of ulna and radius is connected with our upper arm humerus, forming the elbow joint [25]. The olecranon fossa at humerus and the olecranon process at ulna are connected with each other as shown in Figure 2. This connection will limit the range of elbow extension and make the forearm usually in anterior of upper arm. Accordingly, it is reasonable to suppose that the forearm of EEULRebot should also be in one side of its upper arm during all the tasks. Then, we can get the determined inverse kinematic solution calculated as following process.

**Figure 3:**
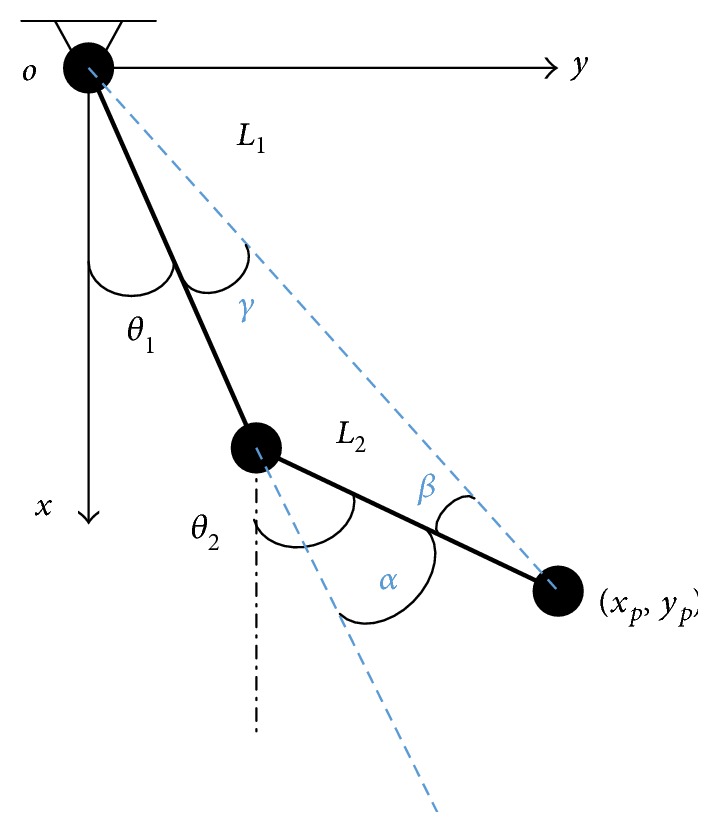
The kinematic model of EEULRebot. *θ*_1_ and *θ*_2_ refer to each motor rotation angle relative to the settled zero reference position (*θ*_1_ = *θ*_2_ = 0 while the two links are parallel to the *x*-axis). *L*_1_ and *L*_2_ mean the length of the EEULRebot upper arm and forearm. The end point position is (*x*_*p*_, *y*_*p*_). *α*,  *β*, and *γ* represent the angles between the segments and the reference lines.

The kinematic model of EEULRebot, as shown in Figure 3. According to the previously mentioned characteristics that the forearm is always in anterior of the upper arm during upper limb movements, we get the solution constraints in inverse kinematics: −*π*/2 ≤ *θ*_1_ ≤ *π*/2, 0 ≤ *θ*_2_ − *θ*_1_ ≤ *π*.

(1) $$\begin{array}{c} \begin{array}{l} \alpha =\theta_{2}-\theta_{1}=\cos^{-1}\frac{{x_{p}}^{2}+{y_{p}}^{2}-{L_{1}}^{2}-{L_{2}}^{2}}{2\times L_{1}\times L_{2}},\\ \beta =\cos^{-1}\frac{L_{1}\cos\alpha +L_{2}}{\sqrt{{x_{p}}^{2}+{y_{p}}^{2}}},\\ \gamma =\alpha -\beta ,\\ \theta_{1}=\cos^{-1}\frac{x_{p}}{\sqrt{{x_{p}}^{2}+{y_{p}}^{2}}}-\gamma ,\\ \theta_{2}=\theta_{1}+\alpha . \end{array} \end{array}$$

Then, the program is implemented in C++ software (Microsoft Visual C++ 6.0) and converse *θ*_1_ and *θ*_2_ to the steps of each motor for rotation function.

### 2.3. Dynamic Model

Since trainings in one training session are always on the supporting surface, which is a fixed plane during the training, the effect of gravitational potential energy can be ignored.

**Figure 4:**
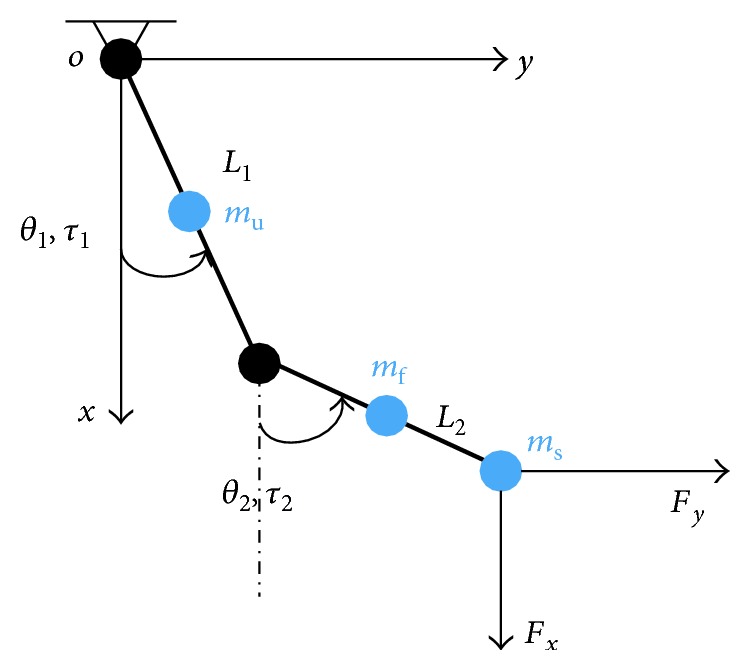
The dynamic model of EEULRebot.

As shown in Figure 4, *θ*_1_, *θ*_2_, *L*_1_, and *L*_2_ represent the same variables as they do in Figure 3. *F*_*x*_ and *F*_*y*_ are the external forces at the end-effector of EEULRebot. *τ*_1_ and *τ*_2_ represent the torque of each motor and *m*_u_, *m*_f_, and *m*_s_, respectively, mean the mass of upper arm, forearm of EEULRebot, and the force sensor (since *m*_s_ is in the same magnitude with *m*_u_ and *m*_f_, we must consider *m*_s_ during dynamic modeling). *τ*_1_ and *τ*_2_ are calculated according to Lagrange's formulation.

Kinetic energy of upper limb is calculated as follows:

(2) $$\begin{array}{c} E_{k}=\left(\frac{1}{6}m_{u}+\frac{1}{2}m_{f}+\frac{1}{2}m_{s}\right){L_{1}}^{2}{\dot{\theta_{1}}}^{2}+\left(\frac{1}{6}m_{f}+\frac{1}{2}m_{s}\right){L_{2}}^{2}{\dot{\theta_{2}}}^{2}+\left(\frac{1}{2}m_{f}+m_{s}\right)L_{1}L_{2}\dot{\theta_{1}}\dot{\theta_{2}}\cos\left(\theta_{1}-\theta_{2}\right). \end{array}$$

We select the training plane as the potential energy zero, *E*_*p*_ = 0.

The Lagrange function is shown as follows:

(3) $$\begin{array}{c} L=E_{k}-E_{p}=\left(\frac{1}{6}m_{u}+\frac{1}{2}m_{f}+\frac{1}{2}m_{s}\right){L_{1}}^{2}{\dot{\theta_{1}}}^{2}+\left(\frac{1}{6}m_{f}+\frac{1}{2}m_{s}\right){L_{2}}^{2}{\dot{\theta_{2}}}^{2}+\left(\frac{1}{2}m_{f}+m_{s}\right)L_{1}L_{2}\dot{\theta_{1}}\dot{\theta_{2}}\cos\left(\theta_{1}-\theta_{2}\right). \end{array}$$

By Lagrange's formulation, the equivalent joint torques can be calculated as follows:

(4) $$\begin{array}{c} \begin{array}{l} \tau_{1e}=\frac{d}{dt}\left(\frac{\partial L}{\partial \dot{\theta_{1}}}\right)-\frac{\partial L}{\partial \theta_{1}},\\ \tau_{2e}=\frac{d}{dt}\left(\frac{\partial L}{\partial \dot{\theta_{2}}}\right)-\frac{\partial L}{\partial \theta_{2}}. \end{array} \end{array}$$

Since the movement of each motor is with small velocity, then, the equivalent joint torque caused by the Coriolis force and centrifugal force can be ignored and the calculation can be simplified as follows:

(5) $$\begin{array}{c} \begin{array}{l} \tau_{1e}=\left(\frac{1}{3}m_{u}+m_{f}+m_{s}\right){L_{1}}^{2}\ddot{\theta_{1}}+\left(\frac{1}{2}m_{f}+m_{s}\right)L_{1}L_{2}\cos\left(\theta_{1}-\theta_{2}\right)\ddot{\theta_{2}},\\ \tau_{2e}=\left(\frac{1}{3}m_{f}+m_{s}\right){L_{2}}^{2}\ddot{\theta_{2}}+\left(\frac{1}{2}m_{f}+m_{s}\right)L_{1}L_{2}\cos\left(\theta_{1}-\theta_{2}\right)\ddot{\theta_{1}}. \end{array} \end{array}$$

The velocity Jacobian of the EEULRebot system can be described following

(6) $$\begin{array}{c} J=\left(\begin{array}{cc} -L_{1}\sin\theta_{1} & -L_{2}\sin\theta_{2}\\ L_{1}\cos\theta_{1} & L_{2}\cos\theta_{2} \end{array}\right). \end{array}$$

Equivalent torque of external forces is calculated following

(7) $$\begin{array}{c} \tau_{e}=J^{T}\left(\begin{array}{c} F_{x}\\ F_{y} \end{array}\right). \end{array}$$

Then, the motor torques can be calculated as follows:

(8) $$\begin{array}{c} \left(\begin{array}{c} \tau_{1}\\ \tau_{2} \end{array}\right)=\left(\begin{array}{c} \tau_{1e}\\ \tau_{2e} \end{array}\right)-J^{T}\left(\begin{array}{c} F_{x}\\ F_{y} \end{array}\right). \end{array}$$

The physical significance of all symbols in the equations is explained in Table 1.

### 2.4. Control Strategy

Hemiplegic patients need different training modes in different conditions [26, 27]. The passive-guided mode is needed while patients are lacking of voluntary movement in early stage after a stroke. When the movement ability is improved, the system can assist the patients to perform training tasks. When patients' abilities are recovered more, the patients will need some challenge in the training tasks [28]. All of these training modes should be included in the control system. Three training modes were implemented in EEULRebot control system: passive-guided mode, active-constrained mode, and active assistant or resistant mode.

Reaching from one point to another point is a basic movement in upper limb movement [29] and tracking a circle is known as the basic movement involving shoulder and elbow joint coordination [30]. Therefore, EEULRebot chooses a point-to-point line tracking and a circle tracking as the training tasks. In order to maximize the range of motion but do not cause discomfort, the two endpoint should be selected by the patients' therapist according to patients' shoulder and elbow passive maximum degrees of freedom.

### 2.5. Passive-Guided Mode

**Figure 5:**
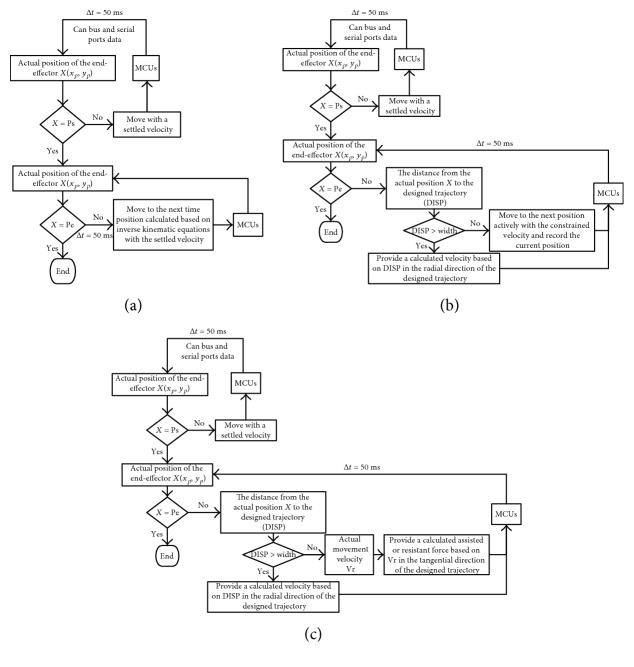
Three control modes of EEULRebot.

During the passive-guided mode, it was the most important thing to move patients' hand in an accurate trajectory to inhibit patients' abnormal movement patterns during the tasks. In this mode, we designed a position loop control with a settled velocity to provide pulses to motor control units (MCUs) based on the inverse kinematic calculation from the current position to the next time position (∆*t* was set as 50 ms for the calculation of motor angles and for movement control information transformed from personal computer to MCUs). The control loop system was shown in Figure 5(a).

### 2.6. Active-Constrained Mode

Active-constrained mode meant a training mode that restricted patients' motion range at the end-effector. Firstly, we designed a motion range along the desired trajectory, called fault tolerance zone (FTZ). In order to make the width of FTZ suitable for the specific patient, the patient should first perform the desired trajectory actively with no constraint. The default width of FTZ was set as 50 mm, and this width value would be updated according to the patient's performance. Once the maximum deviation from the active-with-no-constraint performance to the desired trajectory was smaller than 50 mm, the width should be decreased until it was smaller than the maximum deviation.

The active-constrained mode was designed based on a regional position and velocity loop as shown in Figure 5(b). Once the end-effector was outside of the trajectory and its FTZ, the EEULRebot would provide a helpful motion to move the end-effector back to the region. The helpful motion was designed as a straight line motion from the current point to the point on the desired trajectory, which point made the minimum distance from current point to the designed trajectory.

### 2.7. Active Assistant or Resistant Mode

**Figure 6:**
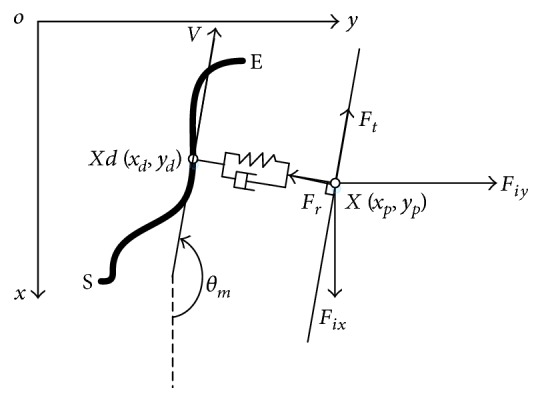
The illustration of impedance control strategy. The bold black curve was the defined trajectory (S and E, resp., represented the start and end point in the designed trajectory); *X* (*x*_*p*_, *y*_*p*_) was the coordinate of the real position; *X*_*d*_ (*x*_*d*_, *y*_*d*_) was the point on the designed trajectory determined by the position which produced the minimum distance from the real position *X* (*x*_*p*_, *y*_*p*_). *V* represented the movement direction at the designed point *X*_*d*_ (*x*_*d*_, *y*_*d*_) in the defined trajectory; *θ*_*m*_ represented the angle between *v* and the *x*-axis; *F*_*t*_ and *F*_*r*_ were, respectively, the required force. *F*_*t*_ was parallel to the direction of *v*, and *F*_*r*_ was perpendicular to *F*_*t*_. *F*_*t*_ was the designed assistant or resistant force calculated based on the actual moment velocity, and *F*_*r*_ was the designed assisted resilience based on the absolute value of distance between X and *X*_*d*_; *F*_*ix*_ and *F*_*iy*_ were the interaction forces detected by the two-dimensional force sensor.

Active assistant or resistant mode was provided for patients who had some voluntary movement ability less than or more than the tracking tasks required. Patients' voluntary movement ability was evaluated by their movement velocity. If the movement velocity was bigger than 50 mm/s, it meant the patient had more voluntary ability than the task required, vice versa [24]. It was also of great importance in this mode to detect the interaction force between EEULRebot and the patient to calculate the assistance or resistance. We designed this mode based on a regional position, velocity, and force loop control shown in Figure 5(c). The desired position and velocity were calculated by inverse kinematic analysis, and the desired force was calculated based on inverse dynamic analysis as well as the impedance-based control theory. Impedance control, proposed by Hogan in 1985, was designed to make the interaction environment between patients and the robot more harmonious [31]. The interaction environment was equivalent as a virtual mass-spring-damp system. The impedance control strategy can be illustrated in Figure 6.

The active assistant or resistant mode was developed based on the active-constrained mode, which was more complicated when the point was within the region of FTZ. If the current point was outside of the FTZ, EEULRebot should rotate the robot upper limb and forearm to provide a radial helpful force *F*_*r*_ to pull the end-effector back into the region of FTZ with *F*_*t*_ = 0. Besides, while the current point was within the region of FTZ, the active assistant or resistant mode should also provide an external force *F*_*t*_ in movement direction according to the movement velocity with *F*_*r*_ = 0. The movement velocity could be calculated based on the angular velocity of each motor and the theorem of composition of velocities. If the movement velocity was smaller than the velocity settled by the therapist, EEULRebot should provide a positive *F*_*t*_ as assistance to help the participant complete the task. The default velocity was set as 10 mm/s which was considered as a velocity that could produce a normal movement. While the movement velocity was bigger than the settled velocity, EEULRebot should provide a negative *F*_*t*_ as resistance to increase the task difficulty for the participant.

The values of *F*_*r*_ and *F*_*t*_ can be calculated based on the impedance control strategy shown in (9)-(10).  *v*_*a*_ meant the actual movement velocity, and *v*_*s*_ meant the settled velocity in the movement task.

(9) $$\begin{array}{c} F_{r}=\left\{\begin{array}{cc} B\left(v_{a}-v_{s}\right)+K∆X & \left(while\;points\;out\;of\;FTZ\;region\right)\\ 0 & \left(while\;points\;in\;region\;of\;FTZ\right), \end{array}\right. \end{array}$$

(10) $$\begin{array}{c} F_{t}=\left\{\begin{array}{cc} 0 & \left(while\;points\;out\;of\;FTZ\;region\right)\\ B\left(v_{a}-v_{s}\right) & \left(while\;points\;in\;region\;of\;FTZ\right). \end{array}\right. \end{array}$$

**Figure 7:**
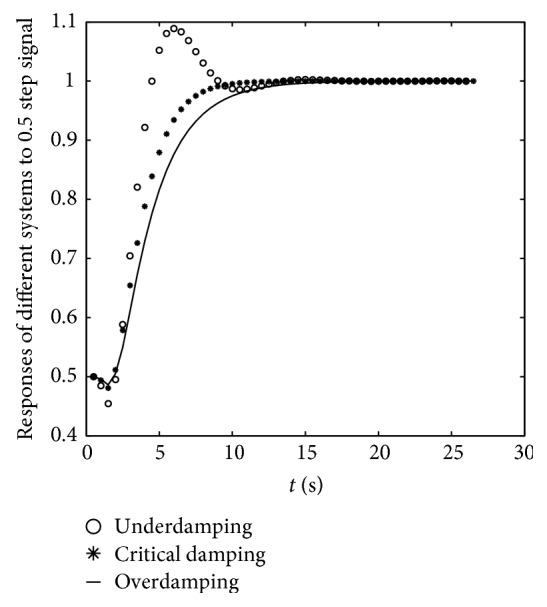
The system response to step signal with amplitude of 0.5 in the environment mass-spring-damp system with different damping coefficients. *x*: the time of response; *y*: response values to 0.5 step signal. The continuous black solid line was the response of overdamping system, and it had no overshoot and a stable value at the end.

It can be found from Figure 7 that overdamping system was better than underdamping and critical damping systems with a stable response to the same step signal. Then, we chose *B* = 2.5 Ns/m and *K* = 1 N/m as a simple overdamping system to make the interaction environment a stable system.

## 3. Experiments and Results

### 3.1. Subjects

In order to demonstrate the usability of EEULRebot, we designed two experiments to test the system movement accuracy and the influence of different movement modes on subjects in passive-guided and active-constrained mode. Eleven healthy subjects (ages: 26.45 ± 9.37, BMI: 22.61 ± 2.97) took part in both the experiments, and three patients (males, ages: 46 ± 16.52, BMI: 25.34 ± 1.36) participated in the passive-guided mode experiment. But only one hemiplegic patient (65 years old, BMI = 23.78) participated in active-constrained mode experiment because other two patients had no voluntary movement ability at the elbow extension. All the participants were provided with the informed consent form before the experiments; the experiments were approved by the Medical Ethics Committee of the Affiliated Hospital of National Research Center for Rehabilitation Technical Aids.

### 3.2. Experiment Process

Each subject was asked to sit in a chair with his/her trunk strapped to restrain his/her trunk moment. The experiment process included two experiments (shown in Table 2): passive-guided mode experiment (PGE) and active-constrained mode experiment (ACE). Participants should perform a line or a circle tracking task with EEULRebot at passive-guided mode during PGE, which asked patients to make no effort to move the end-effector. While during ACE, participants should perform the same task by themselves with constraint. In order to test whether the constraint worked well on the subjects, each participant was asked to perform the same task actively with no constraint at first during ACE. Each subject was asked to perform five trials in each task during both PGE and ACE to reduce the random error.

During PGE, the movement information was collected by the robot encoders. The movement information of robot end-effector was used to describe the accuracy of robot system. During ACE, participants' surface electromyographic signals (EMG) and the interaction force between the robot and participants were recorded. EMG signals of trapezius (TR), pectoralis major (PM), anterior, median and posterior deltoid (AD, MD, and PD), biceps brachii (BB), triceps brachii (TB), and brachioradialis (BR) were recorded by 8-channel Telemyo DTS (Noraxon, USA) with cutoff frequency of 1500 Hz and sample frequency of 1500 Hz. The interaction force was recorded by the two-dimensional force sensor with sample frequency of 5 Hz. The EMG analysis system and the force-recorded system were synchronized by a high level trigger signal with frequency of 100 Hz. EMG signals and the interaction force were used to explore the influence of the constraint on the participants.

### 3.3. Data Processing

#### 3.3.1. Analysis of Movement Accuracy of Robot System

During PGE, the average distance between the actual point and the designed trajectory was calculated according to (11), which was used to describe the movement accuracy during passive-guided mode.

(11) $$\begin{array}{c} {DISP}_{aver}=\frac{\sum_{i=1}^{N}{DISP}_{i}}{N}. \end{array}$$

In (11), DISP_*i*_ meant the distance from actual point *i* to the designed trajectory. *N* meant the number of actual points during the task.

#### 3.3.2. Analysis of Influence of the Different Movement Modes on Healthy Subjects and Patients

The EMG and interaction force were used to explore the influence of the different control modes on the subjects. The raw EMG signals were rectified and reduced the electrocardiogram (ECG) in the commercial software (MR-XP 1.07 Master Edition). Then, the signals were filtered by a bidirectional Butterworth band-pass filter with cutoff values of 10 Hz and 500 Hz in the same software [32–34]. After filtering, the signals were smoothed by calculating the average with a window of 50 ms. The mean EMG (MEMG) in the task duration among 3 trials were averaged to describe each muscle energy in the task. MEMG were normalized by (12), thus making the MEMG comparable between different subjects and different movement modes (with constraint and with no constraint).

(12) $$\begin{array}{c} {MEMG}_{normalized}=\frac{{MEMG}_{i}}{\sum_{i=1}^{n}{MEMG}_{i}}\times 100\%. \end{array}$$

MEMG_*i*_ meant the mean EMG of *i* muscle, *i* = 1, 2, 3,…, 8.

The interaction force and distance from the actual point to the designed trajectory among the task were smoothed and linear interpolation to 100 points and then averaged among 3 trials in the task for one subject performance. The changes among interaction force and distance were compared between different movement modes to describe the influence.

## 4. Results and Discussion

### 4.1. Movement Accuracy of Robot System

The movement accuracy was calculated as the average distance from the actual point to the designed trajectory. The deviation of the passive-guided movement along a designed trajectory was less than 1 mm illustrated in Table 3. Besides, the deviation in patients was not significantly different with that in healthy subjects. The small deviation and difference among different subjects demonstrated the good movement accuracy of the robot system.

### 4.2. The Influence of Control Mode on Interaction Force and Movement Accuracy

**Figure 8:**
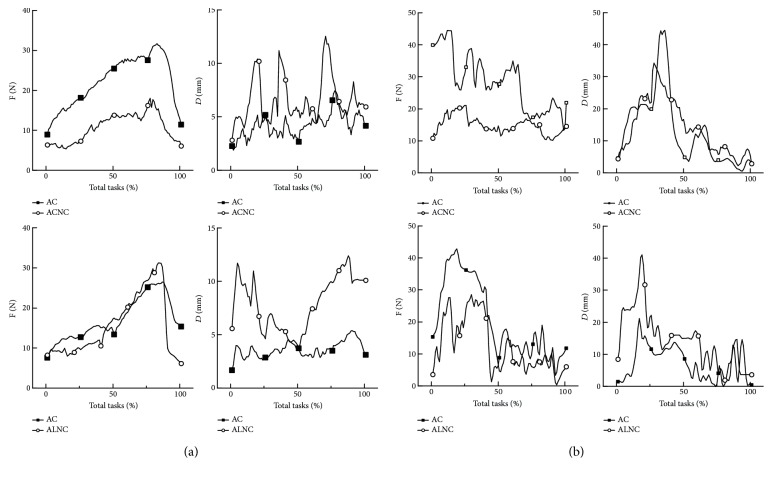
The interaction force and end-effector distance during ACE. (a) The average interaction force and end-effector distance of healthy subjects and (b) that of the hemiplegic patient. *F*: the interaction force; *D*: the distance; AC: active circle tracking task with constraint; AL: active line tracking task with constraint; ACNC: active circle tracking task with no constraint; ALNC: active line tracking task with no constraint.

The forces and displacement were the primary two external factors in human movement. The active movement with no constraint during the ACE was implemented as a comparison test with the active movement with constraint. A circle tracking task or a line tracking forward and backward was normalized by time to be a task with 100 points in the length of total time. The interaction force and distance among different control modes were, respectively, compared with each other (Figure 8).

It can be found that the active-constrained mode movement can bring in a large interaction force with the largest force twice of that in the no-constraint mode movement, especially in the latter part of the task for healthy subjects and in the former part for the patient. Besides, a more accurate movement was achieved by the constrained mode, which can be demonstrated by the smaller distance in Figure 8.

The movement in ACE with constraint was more accurate, because once the end-effector was out of the fault tolerance zone (FTZ) the robot would rotate its arms to bring the end-effector back to the zone. And the movement of robot upper limb and forearm would also increase the interaction force between human and robot. Therefore, the bigger interaction force and the more accurate movement were consistent with each other in the ACE with constraint.

The visual biofeedback may be the factor that caused the big interaction force and the distance of end-effector in the latter part of tasks (the tracking from the farthest point to the nearest point) for healthy subjects. The sight of participants may be blocked by their body and the end-effector handle during the latter part, which revealed the importance of biofeedback in robot therapy and the necessity of the adjustable part in the robot structure. As for the hemiplegic patient, the biggest interaction force and distance were observed in the former part (the tracking from the nearest point to the farthest point). It can be explained by the stereotypic movement pattern between shoulder and elbow joints: shoulder abduction accompanied by elbow flexion [35, 36]. During the former part of tasks, patients should perform shoulder abduction and elbow extension, while the accompanied elbow flexion movement in patients increased the interaction force and the distance.

### 4.3. The Influence of Control Mode on Muscle Activation Distribution

The interaction force was the external factor between human and the robot, and the muscle strength and activation would be the internal factor. Therefore, the EMG of the eight muscles was recorded, which were involved in shoulder external rotation, flexion, and abduction as well as elbow flexion. The normalized mean EMG (MEMG_normalized_) calculated according to (12) was used to describe each muscle effort to complete the task. Besides, several independent *t*-tests were used to analyze the difference of each muscle effort during tasks in healthy subjects. Statistical significance was set at *p* < 0.05.

**Figure 9:**
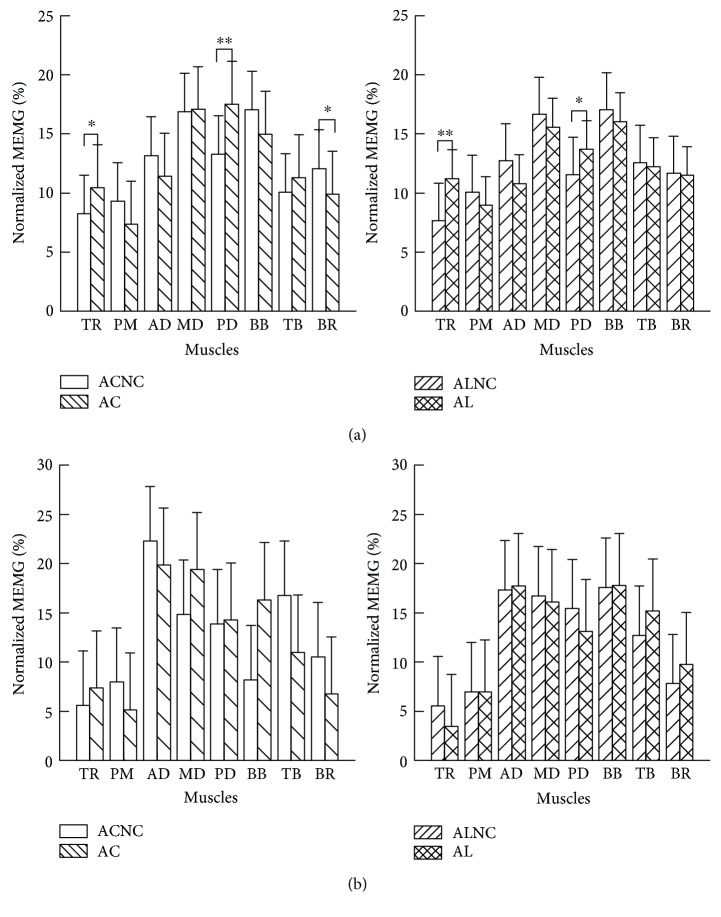
The normalized MEMG of the eight muscles among four actions during ACE. (a) Each muscle effort of healthy subjects during different tasks and (b) that of the only hemiplegic patient. ^∗^0.01 < *p* < 0.05; ^∗∗^*p* < 0.01. The abbreviations (AC, ACNC, AL, and ALNC) were in the same representation with that in Figure 8.

The normalized MEMG of healthy subjects while completing the four tasks (active circle or line tracking with no constraint and with constraint (ACNC, AC, ALNR, and AL)) were shown in Figure 9(a). The effort of each muscle contributing to complete a task was modified by the constraint. The normalized MEMG of TR and PD were significantly larger in tasks with constraint than that in tasks with no constraint both in circle tracking and in line tracking, while the normalized MEMG of PM, AD, BB, and BR were smaller.

The changes of muscle activation distribution in the hemiplegic patient were not the same with that in the healthy subject (founded in Figure 9). The changes of the normalized MEMG of most muscles except BB and TB in circle tracking tasks were the same with that in healthy subjects. However, the change trends of most muscles except MD during line tracking task in the hemiplegic patient were different with that in healthy subjects. The constraint mode had more activation of BB and less TB activation during circle tracking and had more BB and TB activation during line tracking, which was the opposite change trends of the same muscles in healthy subjects. The different changes at elbow flexion and extension muscle group suggest hemiplegic subjects may have much lesion in elbow flexion and extension control ability [37].

Comparing the changes of muscle activation distribution between healthy subjects and patients, it indicated that the active-constrained mode movement can adjust the muscle activation distribution of hemiplegic patients similar to healthy subjects during circle tracking tasks, which suggested that hemiplegic patients innervated muscles in a similar way to healthy subjects. Besides, circle tracking was more variable than the line tracking in the changes of distance of end-effector (shown in Figure 8(b)), which suggested circle tracking had more variability than line tracking. More variability was beneficial for cerebellum development [38]. Moreover, the circle tracking would require more joint movements than line tracking [30, 38], which can contribute to the coordination of shoulder and elbow joints. All above, the circle tracking task in robot active-constrained mode should be a basic training to promote patients' recovery.

## 5. Conclusions

In this article, the new EEULRebot system was developed. The movement accuracy of the system at passive-guided proved the usability of the robot in participants' training. Besides, the influence of active-constrained mode on the participants' interaction force and their internal muscle activation distribution was explored, in which we designed the constraint correlated with the deviation of the actual point to designed trajectory. This study confirmed the constraint at end-effector modified the muscle activation distribution in the same trend in hemiplegic patients and healthy subjects in circle tracking, which suggested that the circle tracking may be a representative motion in rehabilitation training to improve the muscle activation pattern the same with healthy subjects.

The study has demonstrated the usability of passive-guided and active-constraint mode in the experiment. However, the number of the patients is too small. Therefore, we will enroll more hemiplegic patients to complete the experiment in the future. Besides, we will also do experiment on the active assistant or resistant mode to demonstrate its usability on patients.

## Figures and Tables

**Table 1:** The physical significance of all symbols in equations.

Symbols	Physical significance
*θ*_1_	The motor angle of EEULRebot upper arm
*θ*_2_	The motor angle of EEULRebot forearm
*L*_1_	The length of EEULRebot upper arm
*L*_2_	The length of EEULRebot forearm
*m*_u_	The mass of EEULRebot upper arm
*m*_f_	The mass of EEULRebot forearm
*m*_s_	The mass of force senor at the end-effector of EEULRebot
*E*_k_	Kinetic energy
*E*_p_	Potential energy
*L*	Mechanical energy
*τ*_1*e*_	Resultant external torque of motor of EEULRebot upper arm
*τ*_2*e*_	Resultant external torque of motor of EEULRebot forearm
*J*	The Jacobian matrix of the EEULRebot system
*τ*_*e*_	Equivalent torque of external forces
*F*_*x*_	The external force in *x*-axis
*F*_*y*_	The external forces in *y*-axis
*τ*_1_	Motor torque of EEULRebot upper arm
*τ*_2_	Motor torque of EEULRebot forearm

**Table 2:** Participants in the experiment groups and actions.

Experiments	Actions	Healthy subjects	Hemiplegic patients
Passive-guided mode experiment (PGE)	Line tracking and circle tracking task with robot at passive-guided mode	11	3
Active-constrained mode experiment (ACE)	Line tracking and circle tracking tasks actively without constraint (ALNC and ACNC)	11	1
	Line tracking and circle tracking tasks actively with constraint (AL and AC)	11	1

**Table 3:** The deviation of actual trajectory and designed trajectory during passive-guided mode movement.

Action	DISP_aver_/mm (mean ± SD)	
	Healthy subjects (10 men)	Hemiplegic patients (3 men)
Passive-guided line tracking	0.51 ± 0.13	0.39 ± 0.01
Passive-guided circle tracking	0.53 ± 0.32	0.69 ± 0.52
